# Neuroprotection and axon regeneration by novel low-molecular-weight compounds through the modification of DOCK3 conformation

**DOI:** 10.1038/s41420-023-01460-8

**Published:** 2023-05-15

**Authors:** Kazuhiko Namekata, Naoki Tsuji, Xiaoli Guo, Euido Nishijima, Sari Honda, Yuta Kitamura, Atsushi Yamasaki, Masamichi Kishida, Jun Takeyama, Hirokazu Ishikawa, Youichi Shinozaki, Atsuko Kimura, Chikako Harada, Takayuki Harada

**Affiliations:** 1 Visual Research Project, Tokyo Metropolitan Institute of Medical Science, Tokyo, Japan; 2 R&D Division, Daiichi Sankyo Co., Ltd, Tokyo, Japan; 3 Biological Research Department, Daiichi Sankyo RD Novare Co., Ltd, Tokyo, Japan

**Keywords:** High-throughput screening, Optic nerve diseases

## Abstract

Dedicator of cytokinesis 3 (DOCK3) is an atypical member of the guanine nucleotide exchange factors (GEFs) and plays important roles in neurite outgrowth. DOCK3 forms a complex with Engulfment and cell motility protein 1 (Elmo1) and effectively activates Rac1 and actin dynamics. In this study, we screened 462,169 low-molecular-weight compounds and identified the hit compounds that stimulate the interaction between DOCK3 and Elmo1, and neurite outgrowth in vitro. Some of the derivatives from the hit compound stimulated neuroprotection and axon regeneration in a mouse model of optic nerve injury. Our findings suggest that the low-molecular-weight DOCK3 activators could be a potential therapeutic candidate for treating axonal injury and neurodegenerative diseases including glaucoma.

## Introduction

Dedicator of cytokinesis 3 (DOCK3) belongs to the DOCK family of atypical guanine exchange factors (GEFs) and it activates the Rho GTPase Rac1 [1]. DOCK3 is mainly expressed in the brain, spinal cord and retina, and it is the only DOCK protein that shows almost exclusive expression to the central nervous system (CNS) [2, 3]. Deletion of DOCK3 in mice demonstrates axon degeneration and sensorimotor impairments [4]. In humans, loss of function DOCK3 variants causes developmental delay and hypotonia, probably because of abnormal axonal development [5, 6]. DOCK3 is also associated with Alzheimer’s disease (AD), and its expression is reduced from the soluble fraction of AD brain compared with the age-matched controls [7]. On the other hand, overexpression of DOCK3 decreases β-amyloid precursor protein (APP) secretion, which reflect on the acceleration of APP degradation [8]. In addition, DOCK3 stimulates cytoskeletal remodeling through GEF-dependent and GEF-independent pathways [9] and induces axon regeneration after optic nerve injury [3]. Furthermore, overexpression of DOCK3 promotes neuroprotection in a mouse model of glaucoma [10].

One of the typical signaling pathways in which DOCK3 stimulates neurite outgrowth is the Engulfment and cell motility protein 1 (Elmo1)-RhoG pathway. DOCK family proteins interact with Elmo1 in the cytoplasm, and the interaction is required for the biological function [11, 12]. For example, DOCK3 is recruited to the plasma membrane by forming a DOCK3-Elmo1-RhoG complex, leading to translocation of WASP (Wiskott–Aldrich syndrome protein) family verprolin-homologous (WAVE) protein, Rac1 activation, and actin dynamics [13]. These findings prompted us to find novel DOCK3 activators that enhance the interaction between DOCK3 and Elmo1. In this study, we synthesized and screened low-molecular-weight compounds for DOCK3 activators, and examined the lead compounds for their effects on neuroprotection and axon regeneration in a mouse model of axon injury.

## Results

### High throughput screening for a potent DOCK3 activator

**Fig. 1:**
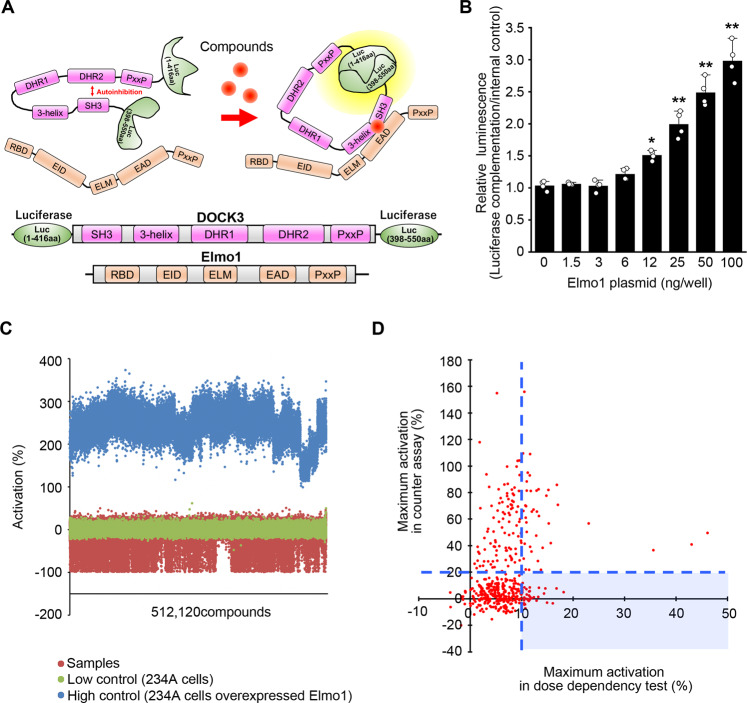
Screening of DOCK3 activators. **A** Schematic diagram of the luciferase complementation assay. The N-terminal fragment (1-416aa) and the C-terminal fragment (398–550aa) of luciferase were connected to the N- and C-terminal sides of DOCK3, respectively. It is assumed that hit compounds release the autoinhibition of DOCK3 by stabilizing the interaction between DOCK3 and Elmo1, which causes the luciferase fragments to associate and the signal from the chemiluminescent reaction is detected. **B** Validation of the luciferase complementation assay. Signal enhancement is observed depending on the concentration of the introduced Elmo1 gene. *n* = 4 well per group. Asterisk depicts statistically significant changes compared to DMSO control (0 ng/well). **P < 0.01; *P < 0.05. Data represent the mean ± S.E.M. **C** The scatter plot indicates that there was a robust assay signal window between the low control (no treatment; brown) and high control (Elmo1 overexpression; blue) and that the majority of compounds (samples; green) were inactive and exhibited activity levels that coincided with the low controls. **D** Correlation between dose dependency test and counter test. Each spot represents the maximum value of the mean of activation (%) in the dose dependency test or counter test. Compounds in the light blue area were selected. After confirmation of the dose dependency, 14 compounds that met the criteria were processed for the next assay.

**Fig. 2:**
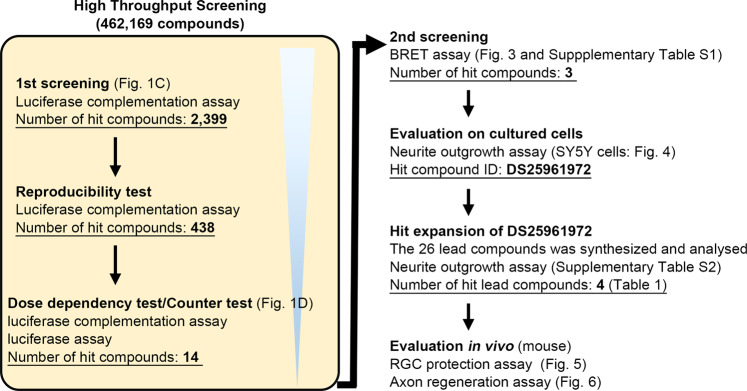
Summary of the high throughput screening of DOCK3 activators. One hit compound was selected from 462,169 compounds and four hit lead compounds were processed for in vivo evaluation.

In order to screen for low-molecular-weight compounds that enhance the DOCK3 GEF activity, we first established a luciferase complementation assay to monitor DOCK3 intramolecular interactions in the presence of Elmo1. SH3 domain in DOCK-A (DOCK1, 2, 5) and DOCK-B (DOCK3, 4) interacts with DHR2 domain to maintain the protein in an autoinhibited state, and the interaction is suppressed in the presence of Elmo1 [14–16]. In our assay, the intramolecular interaction of SH3 domain and DHR2 domain within DOCK3 sterically prevents the rearrangement of the functional luciferase reporter molecule (Fig. 1A) [17]. If a small molecule affects the intermolecular interaction between DOCK3 and Elmo1, the release of the autoinhibition will allow the reconstruction of a luciferase reporter molecule. To confirm this point, we first examined the luciferase activity of DOCK3 with various concentration of Elmo1, and found that signal enhancement is dependent on the concentration of the introduced Elmo1 (Fig. 1B). These findings suggest that our system is available for the screening of potent DOCK3 activators. Therefore, we evaluated 462,169 compounds in the low-molecular-weight compound library using the assay system as high throughput screening (Fig. 2).

Quality control for this high throughput screening (HTS) of luciferase complementation assay was ensured using z-value and signal to noise ratio (S/N ratio) for evaluating the suitability of the HTS assay [18]. The evaluation values of the 462,169 compounds are plotted (Fig. 1C). The average of z-values and S/N ratios for all plates were 0.65 ± 0.05 and 13.8 ± 1.9, respectively. As the results of HTS screening, 2,399 compounds met the criteria for hit compounds (Fig. 2). Among them, 438 compounds passed the reproducibility test of HTS screening. For validation of HTS hits, dose dependency test and counter test were conducted with 438 primary hit compounds. This validation process eliminated false positive compounds that were affected by promoters or luminescence reactions. The validation studies identified 36 compounds. These compounds indicated less than 20% of maximum activation in counter test and over 10% of maximum activation in dose dependency test (Fig. 1D). In addition, we selected 14 compounds with most high dose dependency from 36 compounds as primary hit compounds (Fig. 2).

### Secondary screening of the primary hits

**Fig. 3:**
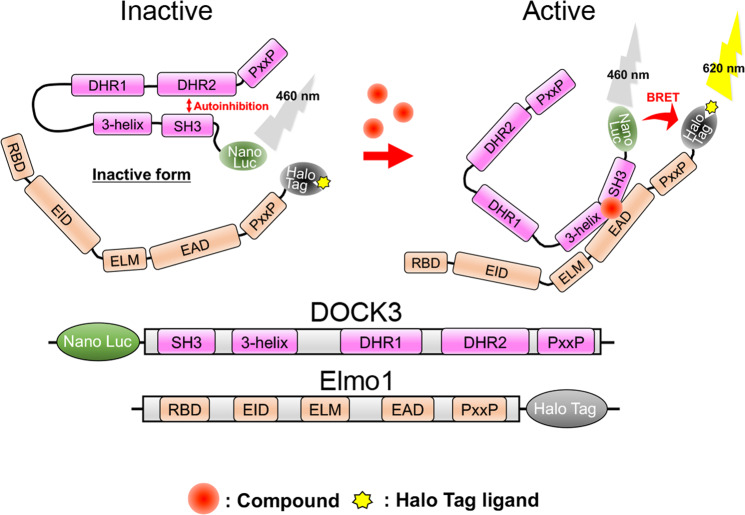
Establishment of intermolecular BRET assay. The association of DOCK3 and Elmo1 shortens the intermolecular distance between Nano Luc and Halo Tag, resulting in the BRET signal detection.

Next, in order to evaluate the interaction of a DOCK3 molecule with Elmo1, we established intermolecular bioluminescence resonance energy transfer (BRET) assay (Fig. 3). BRET is a mechanism for energy transfer between a luminescent molecule (Nano Luc) and a photosensitive molecule (Halo Tag), and this phenomenon can be used to evaluate intermolecular protein interactions. Using this evaluation system, we investigated the 14 primary hit compounds and found that several compounds enhanced BRET signal in a concentration-dependent manner (Supplementary Table 1). Thus, we finally selected top three compounds at high concentration (DS18510010, DS25961972, and DS47590519) as the hit compounds (Fig. 2) because they have the strong capacity to increase the interaction between DOCK3 and Elmo1.

### Effects of the three hit compounds on neurite outgrowth

**Fig. 4:**
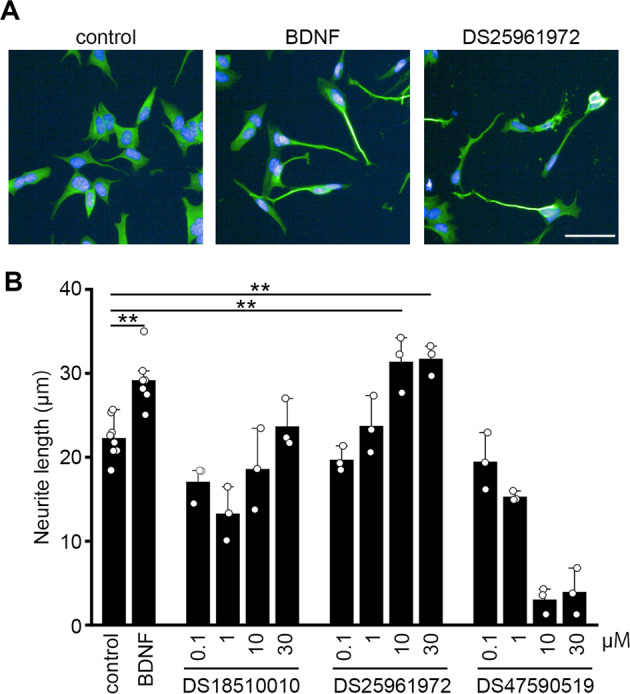
Induction of neurite outgrowth by DS25961972. **A** Representative images of SH-SY5Y cells treated with DMSO (control), BDNF (100 ng/mL), and DS25961972 (30 µM). **B** The neurite length per cell of SH-SY5Y cells treated with various concentrations of DS18510010, DS25961972, and DS47590519. *n* = 3 cells per group. **P < 0.01. Data are expressed as mean ± S.E.M.

We previously reported that overexpression of DOCK3 stimulates neurite outgrowth [3]. We next examined the effects of the three compounds on neurite outgrowth in SH-SY5Y cells. Among the three hit compounds, DS25961972 stimulated neurite outgrowth in a concentration-dependent manner and the effect was similar to brain-derived neurotrophic factor (BDNF) (Fig. 4). Thus, we focused on DS25961972 as the most effective hit compound (Fig. 2).

### Hit expansion of DS25961972 to obtain lead compounds

The 26 derivatives were developed from the hit compound DS25961972 in order to obtain lead compound (Supplementary Table 2). Derivatives were evaluated for neurite outgrowth assay in SH-SY5Y cells or primary cultured cerebellum neurons. We selected four lead compounds (DS76180860, DS59060620, DS72062539, and DS94240266) that was top two compounds in the neurite outgrowth assay of SH-SY5Y cells (DS59060620 and DS72062539) or cerebellum neurons (DS76180860 and DS94240266) (Supplementary Fig. 1). The molecular structures and GEF activities of these four lead compounds were indicated in Table 1.

**Table 1:** Molecular structure and the functions of the four lead compounds of DS25961972.

Compound ID	DS76180860	DS59060620	DS72062539	DS94240266
Neurite outgrowth in SH-SY5Y cells (% of control)	46.8	199.8	144.5	60.3
Neurite outgrowth in cerebellum neurons (% of control)	223.1	145.5	208.6	245.2
GEF activity (% of control)	141.1	158.2	224.4	187.9
Structure				

Since DS72062539 showed the highest GEF activity, we examined whether DS72062539 can stimulate the interaction between DOCK3 and Elmo1 by biochemical assays. Pull-down assay revealed that DS72062539 increases the formation of DOCK3-Elmo1 complex in Cos7 cells (Supplementary Fig. 2).

### Lead compounds for DOCK3 activators demonstrate neuroprotective effects on retinal neurons after optic nerve injury

**Fig. 5:**
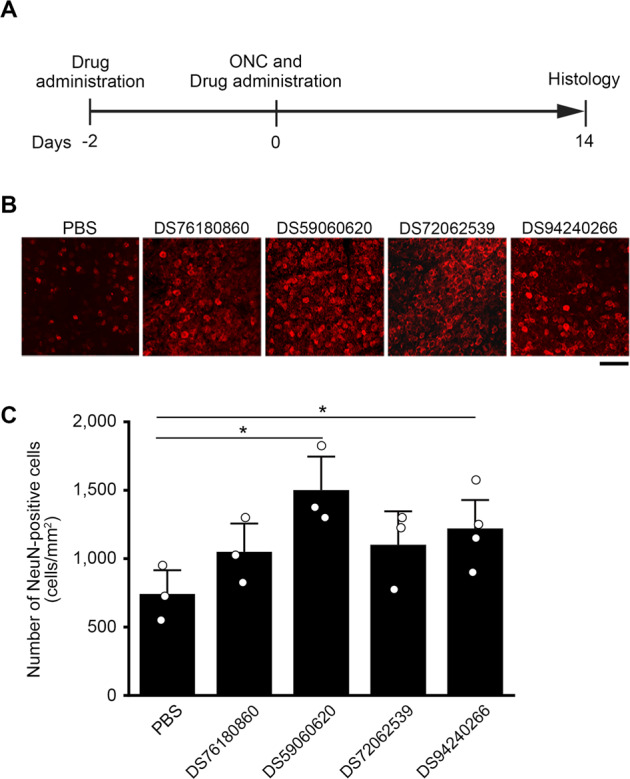
Effects of intravitreal injection of DOCK3 activators on retinal neurons following ONC. **A** Experimental protocol. DOCK3 activators (2 µl) or PBS (2 µl) was administered at 2 days before ONC and 5 min after ONC. Mice were euthanized at 14 days after ONC. **B** Representative images of NeuN-positive cells in the flat mount retina. Scale bar: 50 µm. **C** Quantification of NeuN-positive cells. *n* = 3–4 mice per group. *P < 0.05. Data are expressed as mean ± S.E.M.

We next examined the therapeutic potential of the selected four lead compounds. For this, we used an optic nerve crush (ONC) mouse model. We injected each of the four lead compounds intravitreally twice, one at two days before ONC and the other at 5 min after ONC, and the retinas were examined for neuronal survival two weeks after ONC (Fig. 5A). Retinal flat mounts were immunostained with an anti-NeuN antibody for detection of retinal neurons in the ganglion cell layer for analysis of neuroprotective effect (Fig. 5B). Quantitative analysis demonstrated that the survival of retinal neurons was higher in the retinas treated with DS59060620 and DS94240266 compared with vehicle treated retinas (Fig. 5C). There was a tendency towards increased survival in retinas with DS76180860 and DS72062539, but it was not statistically significant. These data show that these lead compounds exert neuroprotective effects in an ONC model.

### DOCK3 activators promote axon regeneration in an ONC model

**Fig. 6:**
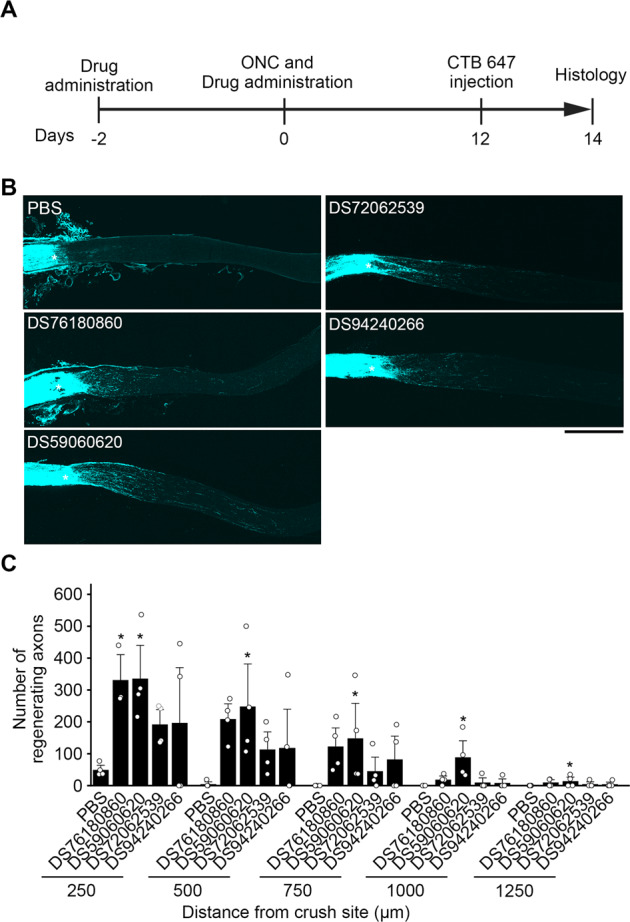
Effects of intravitreal injection of DOCK3 activators on optic nerve regeneration following ONC. **A** Experimental protocol. DOCK3 activators (2 µl) or PBS (2 µl) was administered at 2 days before ONC and 5 min after ONC. To detect regenerating axons, CTB 647 was intravitreally injected after 12 days of ONC, and the mice were euthanized at 14 days after ONC. **B** Representative images of the regenerating axons. Longitudinal sections through the optic nerve showing CTB 647-positive axons 14 days after ONC. Scale bar: 500 µm. **C** Quantitative analyses of the number of regenerating axons extending at 250, 500, 1000, and 1250 µm from the injury site. *n* = 3–4 mice per group. *P < 0.05. Data are expressed as mean ± S.E.M.

Overexpression of DOCK3 induces axon regeneration after ONC in vivo [3]. Therefore, we examined the ability of these lead compounds to promote axon regeneration in an ONC model. The same drug administration protocol was used, and we injected Alexa647-labeled cholera toxin B subunit (CTB 647) intravitreally at 2 days before sample collection for visualizing the regenerating axons (Fig. 6A). Two weeks after ONC, increased number of regenerating axons was observed with treatment with DS76180860 and DS59060620 compared with vehicle (Fig. 6B, C). In particular, in DS59060620-treated eyes, some regenerated axons elongated over 1000 µm from the crush site, whereas no regenerating axon was detected at the corresponding area in control mice. These data demonstrate that these lead compounds are able to promote optic nerve regeneration and suggest that, from our screening compounds, DS59060620 may be the most potent drug for neuroprotection and axon regeneration.

## Discussion

In the present study, we developed a first DOCK3 activator by screening 462,169 compounds in the low-molecular-weight compound library (Fig. 2). The derivative of the best compound could stimulate neuroprotection and axon regeneration after optic nerve injury, and the effect was similar to DOCK3 overexpression in mice [3]. In this study, we selected the compounds that stimulate the interaction between DOCK3 and Elmo1, which lead to enhanced GEF activity and neurite outgrowth. Recent study demonstrated that the DOCK2−Elmo1 complex adopts a closed, auto-inhibited conformation [12]. Conformational change of the Elmo1 subunit relieves the autoinhibition to an active, open state, which exposes binding sites for Rac1 on the DHR2 domain of DOCK2, RhoG, and Elmo1. The basic structure of DOCK3 and DOCK2 are partially similar (52.2%), but the detailed spatial structures of DOCK3 and DOCK3-Elmo1 complex are not validated. Thus, further studies are required to detect the precise mechanism how DOCK3 activators affect the interaction between DOCK3 and Elmo1. In addition, DOCK3 is activated by multiple pathways. For example, BDNF stimulates the formation of a protein complex of DOCK3, the tyrosine kinase Fyn, and WAVE proteins at the plasma membrane, and subsequent Rac1 activation may promote actin polymerization [3] and neuroprotection [19–21]. It is also demonstrated that DOCK3 binds to and inactivates glycogen synthase kinase-3β (GSK-3β) at the plasma membrane, and increases the nonphosphorylated active form of collapsin response mediator protein-2 (CRMP-2), which promotes microtubule assembly. Interestingly, the interaction between DOCK3 and GSK-3β is independent of the GEF catalytic activity, but it can also stimulate axon regeneration. Thus, more effective DOCK3 activators may be detected by different screenings that are focusing on the mechanism other than the association with Elmo1. Recent studies have shown that GSK-3β may be a common therapeutic target for treatment of multiple conditions [22–24]. Novel compounds that affect the association between DOCK3 and GSK-3β may be useful for treating these disorders.

During the screening process, we focused on Elmo1 as a binding partner of DOCK3. Elmo1 is known to bind to the SH3 domain of DOCK1–4 [12, 25, 26]. Thus, activators or inhibitors for DOCK1, 2, and 4 may be detected by a similar screening method. These proteins may be involved in the pathogenesis of cancer, AD, autism, schizophrenia, and so on [27–30]. Thus, activators or inhibitors of DOCK1–4 may be useful for various diseases other than CNS degeneration, such as COVID-19-associated diseases [31]. In addition, we recently demonstrated that DOCK8 and DOCK10 may play important roles in mouse models of neuroinflammation [32, 33]. Loss of function in DOCK8 may induce severe combined immunodeficiency in humans [34]. Although DOCK8 and DOCK10 do not have SH3 domain, screenings of low-molecular-compounds that alter the activity of DOCK family proteins may be intriguing.

One important point in our study is that DOCK3 activator was effective via intraocular injection. Recently, intraocular injection of anti-VEGF agents to prevent neovascularization is usual worldwide for the treatment of diabetic retinopathy, age-related macular degeneration, etc. On the other hand, we recently developed a AAV vector which can prevent the progress of neurodegeneration in mouse models of glaucoma [35]. However, gene therapy is not yet common and there are many restrictions for routine medical care. Considering the situation, low-molecular-weight DOCK3 activator may be useful to treat retinal degenerative disorders including glaucoma. Current therapy for glaucoma is to reduce intraocular pressure by eye drops and filtration surgery, but this strategy is insufficient in more patients than previously expected [36, 37]. Since glaucoma is the second leading cause of blindness in the world, and the number of patients is increasing in the aging society [38], other low-molecular-weight compounds that have neuroprotective effects may be useful in combination with DOCK3 activator. Further studies are required to examine the duration of the effect of DOCK3 activators in the CNS as well as in the eyes.

## Materials and methods

### Cells culture

In all, 293 A cells (Thermo Fisher Scientific, Waltham, MA) were cultured in DMEM containing 10% FBS and 1% penicillin-streptomycin. Transient transfection in 293 A cells was performed using FuGENE HD (Promega, Madison, WI) according to the gene transfer protocol (MaxCyte GT; MaxCyte, Gaithersburg, MD). For neurite outgrowth assay, SH-SY5Y cells (Thermo Fisher Scientific) were cultured in DMEM/F12 medium containing 1% FBS, 1% Penicillin-Streptomycin and 7.5 µM retinoic acid. For purification of recombinant protein, Sf9 cells were cultured in Grace’s Insect Medium.

### Cerebellar neurons culture

A primary culture of cerebellar neurons was prepared from embryonic day 18 mice. Embryos were removed from a pregnant female mouse and dissected in HBSS. After removing the meninges, the intact isolated cerebellar cortex was transferred to a 15-ml Falcon tube containing HBSS. Tissues were digested enzymatically in Accutase solution (Innovative Cell Technologies, San Diego, CA) with 0.02% DNase I (Roche, Basel, Switzerland), incubated at 37 ^o^C for 5 min, and rinsed with Mg^2+^- and Ca^2+^-free HBSS (Gibco-Invitrogen, Grand Island, NY). Following centrifugation at 1500 rpm for 5 min at room temperature. After carefully removing the supernatant, the pellet was resuspended in 1 ml of Neurobasal culture medium supplemented with B-27, 2 mM GlutaMax, 5% horse serum, 5% fetal bovine serum, and a mix of penicillin/streptomycin (1000 U/ml and 100 mg/ml, respectively). Cerebellar neurons were cultured on glass coverslips, which had been coated overnight with a solution of 0.1 mg/ml poly-d-lysine in PBS.

### Luciferase complementation assay

We constructed DOCK3-Luc-comp plasmid and Flag-Elmo1 plasmid for this assay. DOCK3-Luc-comp plasmid expressed a fusion protein, two parts of Firefly luciferase (1-416aa) and (398-550aa) fused at N- and C-terminus of DOCK3, respectively. Flag-Elmo1 plasmid expressed Flag-tagged human Elmo1. The test compound or DMSO was dispensed into 384-well microplates (Corning, Corning, NY) in 100 nl/well (final concentration 5 mg/ml). After transfection with two plasmids (DOCK3-Luc-comp and Flag-Elmo1), cells were prepared to 1.25 × 10^5^ cells/mL using Opti-MEM (Thermo Fisher Scientific) and dispensed at 2.5 × 10^3^ cells/well onto 384-well plates containing the compound. The plates containing the cells were cultured at room temperature for 3 h. After incubation, the plate was added with Bright-Glo (5 µl/well; Promega), stirred, and then centrifuged in order to detect luminescence. Luciferase emission (556 nm) was measured with a ViewLux uHTS Microplate Imager (PerkinElmer, Waltham, MA).

The activation rate of luciferase activity [denoted as Activation (%)] by the compound was calculated by the following formula:

$$\begin{array}{l}{{{\mathrm{Activation}}}}\left( {{{\mathrm{\% }}}} \right){{{\mathrm{ = }}}}\left( {{{{\mathrm{Measured}}}}\;{{{\mathrm{value}}}}\;{{{\mathrm{of}}}}\;{{{\mathrm{Sample}}}}\;{{{\mathrm{well - Average}}}}\;{{{\mathrm{value}}}}\;{{{\mathrm{of}}}}\;{{{\mathrm{Low}}}}\;{{{\mathrm{control}}}}} \right)\\{{{\mathrm{/}}}}\left( {{{{\mathrm{Average}}}}\;{{{\mathrm{value}}}}\;{{{\mathrm{of}}}}\;{{{\mathrm{Low}}}}\;{{{\mathrm{control}}}}} \right){{{\mathrm{ \times 100}}}}\end{array}$$

Z-value and S/N ratio were calculated by the following formulas:

$$\begin{array}{l}{{{\mathrm{Z - value = 1-3 \times }}}}\left( {{{{\mathrm{Low}}}}\;{{{\mathrm{control}}}}\;{{{\mathrm{standard}}}}\;{{{\mathrm{deviation + High}}}}\;{{{\mathrm{control}}}}\;{{{\mathrm{standard}}}}\;{{{\mathrm{deviation}}}}} \right)\\{{{\mathrm{/}}}}\left( {{{{\mathrm{Average}}}}\;{{{\mathrm{value}}}}\;{{{\mathrm{of}}}}\;{{{\mathrm{High}}}}\;{{{\mathrm{control - Average}}}}\;{{{\mathrm{value}}}}\;{{{\mathrm{of}}}}\;{{{\mathrm{Low}}}}\;{{{\mathrm{control}}}}} \right)\end{array}$$

$${{{\mathrm{S/N}}}}\;{{{\mathrm{ratio = }}}}\left( {{{{\mathrm{mean}}}}\;{{{\mathrm{value}}}}\;{{{\mathrm{of}}}}\;{{{\mathrm{Low}}}}\;{{{\mathrm{control}}}}} \right){{{\mathrm{/}}}}\left( {{{{\mathrm{standard}}}}\;{{{\mathrm{deviation}}}}\;{{{\mathrm{of}}}}\;{{{\mathrm{Low}}}}\;{{{\mathrm{control}}}}} \right)$$

Low control: compound-free wells with 293 A cells

High control: compound-free wells with 293 A cells co-expressing Elmo1

Sample well: compound-treated wells with 293 A cells

The selection criteria for luciferase complementation assay were set in consideration of the variability of the evaluation system in order to get the appropriate hit rate. Specifically, referring to the fact that the mean ±3 S.D. of negative control was 22.08, we selected compounds with an Activation (%) of 22 or more. In addition, we rescued 555 compounds that did not reach the activity criterion but had druggable physicochemical properties.

### Reproducibility test

The reproducibility test was performed in the same way as the 1st screening, except that the number of samples was changed from *n* = 1 to *n* = 3. This reproducibility test was performed three times for 2399 compounds (Fig. 2). The selection criteria for the reproducibility test were set in consideration of the variability of the evaluation system so that even weakly active compounds would not be dropped. Specifically, referring to the fact that the mean ± S.D. of negative control was 7.3, we selected compounds with an Activation (%) of 10 or more. We set a low hurdle for the criteria and decided to screen compounds that meet the criteria in subsequent dose-dependent and counter tests.

### Dose dependency test

In the dose-dependent test, the highest evaluation concentration was set to 25 mg/ml or 50 µM, and a 4-step diluted compound solution with a common ratio of 2 was prepared from the highest evaluation concentration. Subsequent assay methods were carried out in accordance with luciferase complementation assay described above except sample size (*n* = 3) (Fig. 2). Whether there was a trend between concentration and activity was visually determined from the graph.

### Counter test

The counter assay was performed using 293A cells into which the two constructs had been introduced. With these cells, false-positive compounds can be eliminated by measuring the luminescence of the Firefly luciferase and the Renilla luciferase (Promega). In particular, one assay excluded compounds that affect promoter activity by measuring the activity of Renilla luciferase driven by the same promoter as in the DOCK3 luciferase complementation assay. The other assay eliminated compounds that enhanced the complementation of split luciferase protein fragments by measuring the activity of another luciferase complementation assay for detecting ETV6 polymerization. For counter assay, we constructed three expression plasmids, FlucN-ETV6, ETV6-FlucC, and Renilla. FlucN-ETV6 plasmid expresses a fusion protein, Firefly luciferase (1-416aa) was fused at N-terminus of human ETV6 (2-320aa). ETV6-FlucC plasmid expresses a fusion protein, human ETV6 (1-140aa) was fused at N-terminus of Firefly luciferase (395-550aa). Renilla plasmid expresses Renilla luciferase. FlucN-ETV6, ETV6-FlucC, and Renilla luciferase-expressing cells were dispensed at 2.5 × 10^3^ cells/well onto 384-well plates containing the compound at the same concentration as the concentration-dependent test. The plates were cultured at room temperature for 3 h. After incubation, the plate was added with Dual-Glo Luciferase Reagent (5 µl /well; Promega), stirred, and then centrifuged in order to detect luminescence. Firefly luciferase emission (556 nm) was measured with a PHERAstar FS (BMG Labtech, GmbH, Germany). Dual-Glo Stop & Glo Reagent (Promega) was applied to each well of the plate after Firefly luminescence measurement at 5 µl/well, and Renilla luciferase luminescence (480 nm) was measured using PHERAstar FS. The activation rate of Firefly luciferase activity or Renilla luciferase activity [expressed as Activation (%)] by compound was calculated by the following formula:

$$\begin{array}{l}{{{\mathrm{Activation}}}}\left( {{{\mathrm{\% }}}} \right){{{\mathrm{ = }}}}\left( {{{{\mathrm{Measured}}}}\;{{{\mathrm{value}}}}\;{{{\mathrm{of}}}}\;{{{\mathrm{Sample}}}}\;{{{\mathrm{well - Average}}}}\;{{{\mathrm{value}}}}\;{{{\mathrm{of}}}}\;{{{\mathrm{Control}}}}} \right)\\{{{\mathrm{/}}}}\left( {{{{\mathrm{Average}}}}\;{{{\mathrm{value}}}}\;{{{\mathrm{of}}}}\;{{{\mathrm{Control}}}}} \right){{{\mathrm{ \times 100}}}}\end{array}$$

Control: compound-free wells with transgenic 293A cells

Sample well: compound-treated wells with transgenic 293A cells

In the counter assay, compounds with activation of 20% or more, which is equivalent to the criteria of the primary screening, were judged to be false positives. Further evaluation was performed on 14 compounds that met the criteria of HTS screening (Fig. 2).

### BRET assay

To evaluate the intermolecular interaction between DOCK3 and Elmo1 by BRET assay, we constructed Nano Luc DOCK3 plasmid (Nano Luc was fused at N-terminus of DOCK3) and Elmo1 Halo Tag plasmid (Halo Tag was fused at C-terminus of Elmo1). Each plasmid was transfected into 293A cells using FuGENE HD. The transfected cells were collected and resuspended in Opti-MEM without phenol red containing 100 nM HaloTag NanoBRET 618 Ligand (Promega), and then transferred into 384-well plates (8000 cells/well) to which the compound had been added beforehand, and the plates were incubated for 4 h in a CO_2_ incubator (37 °C, 5%). In all, 10 µl of substrate (final, 10 µM) dissolved in Opti-MEM without phenol red were added to cells. Thirty min after addition of the substrate, donor emission (wavelength at 460 nm) and acceptor emission were measured by PHERAstar or EnVision (PerkinElmer). Transfection plasmid mixture used are as follows: For high control wells and compound wells, equivalent Nanoluc-DOCK3 plasmid, Elmo1-Halo Tag plasmid and pcDNA3.1 plasmid were mixed. For low control wells, equivalent Nanoluc-DOCK3 plasmid, Elmo1-Halo plasmid and FLAG-Elmo1 plasmid were mixed.

### Neurite outgrowth assay

SH-SY5Y cells were seeded in 384-well plates at 8 × 10^2^ cells/well, incubated for 15 h, and then replaced with DMEM/F12 medium containing 7.5 µM retinoic acid, 1% FBS and 1% penicillin-streptomycin for 24 h. The medium was further changed twice, every 24 h. The medium was then replaced with the compound-containing medium and incubated for 48 h. After removing medium, the cells were fixed by adding 4% PFA for 15 min. Each well was washed with PBS, added 3% BSA, and incubated for 60 min at 37 °C. βIII Tubulin antibody as primary antibody (MAB1195; R&D Systems, Minneapolis, MN) and Alexa Fluor 488 goat anti-mouse IgG as secondary antibody (A-11001; Thermo Fisher Scientific) were used to stain the cells. Cell axon length was measured using Opera Phenix (PerkinElmer).

Primary cerebellum neurons were seeded in 96 well plates at 1 × 10^4^ cells/well, cultured for 24 h. The medium was then replaced with the compound-containing medium and incubated for 48 h. After removing medium, the cells were fixed by adding 4% PFA for 15 min. Each well was washed with PBS, added 3% BSA, and incubated for 60 min at 37 °C. Tau1 antibody as primary antibody (MAB3420A4; Merck/Sigma-Aldrich, Munich, Germany) and Alexa Fluor 488 goat anti-mouse IgG as secondary antibody (A-11001; Thermo Fisher Scientific) were used to stain the cells. Cell axon length was measured using Opera Phenix (PerkinElmer).

### GEF assay

The recombinant hElmo1 used for the GEF activity assay was purified from E. coli expressing His-tagged hElmo1 using Ni-NTA affinity chromatography and size-exclusion chromatography. Recombinant hElmo1 was purified from Sf9 cells co-expressing His- and Flag-tagged DOCK3 using Ni-NTA affinity chromatography, ANTI-FLAG M2 affinity chromatography, and PD-10 Desalting Columns (Merck/Sigma-Aldrich). DOCK3 activity was determined according to the protocol provided with the GTPase-Glo™ Assay (Promega). 384-well plates were pre-incubated with GEF Buffer containing 0.05 µM recombinant hDOCK3 and 0.05 µM recombinant hElmo1 in the presence of DMSO or 0.01, 0.1, 1, and 10 µM of the compounds at room temperature for 30 min. Then, 2 µM Rac1 and 1 µM Rho GAP were added to initiate the GTPase reaction and incubated at room temperature for 2 h. GTPase-Glo Reagent was then added to the completed GTPase reaction, and 30 min later Detection Reagent was added. Luminescence was measured using a microplate reader (SpectraMax; Molecular Devices, Sunnyvale, CA).

### Pull-down assay

After 24 h of transfection with myc-GST-hElmo plasmid, Cos7 cell lysate was incubated with Glutathione Sepharose 4B (GE Healthcare, Chicago, IL) for affinity purification of myc-GST-Elmo. After being washed, the precipitant was subsequentially incubated with Cos7 cell lysate expressed myc-tagged hDOCK3 in the presence or absence of DS72062539 (10 µM) for 10 min at 4°C. After being washed, precipitated samples were subjected to SDS-PAGE followed by immunoblot analysis. Both myc-hDOCK3 and myc-GST-Elmo were detected by anti-myc antibody.

### Animals

Experiments were performed using C57BL/6J mice (CLEA Japan, Tokyo, Japan) in accordance with the ARVO Statement for the Use of Animals in Ophthalmic and Vision Research. Animal experiments were approved by the institutional animal care and use committee of the Tokyo Metropolitan Institute of Medical Science (approval number TMiMS: 18041). No statistical methods were used to predetermine sample sizes. Sample sizes are similar to those used in the field.

### Optic nerve crush

Mice (C57BL/J, male, 8 weeks-old) were anesthetized with isoflurane during ONC. The optic nerve was exposed intraorbitally and crushed at ~0.5–1.0 mm from the posterior pole of the eyeball with fine surgical forceps for 5 s [3, 32]. Mice were randomly divided into 5 groups and received intravitreous injections of DOCK3 activators (DS76180860, DS59060620, DS72062539, DS94240266; 2 µl at 10 µM): 2 days before ONC and 5 min after ONC.

### Quantification of retinal neurons

Mice were perfused with ice-cold PBS, followed by 4% PFA in 0.1 M phosphate buffer (pH 7.4). After perfusion, eyes were enucleated and postfixed in 4% PFA for 2 h, and retinas were isolated for whole-mount preparation. Retinas were immunostained with an anti-NeuN antibody (MAB377; Merck Millipore, Burlington, MA) and an Alexa Fluor 568 conjugated anti-mouse secondary antibody (A-1104; Thermo Fisher Scientific). After immunostaining, retinas were mounted on a glass slide with a mounting medium (Vectashield, Vector Laboratories, Burlingame, CA), and the number of NeuN-labeled neuron in the ganglion cell layer was examined with a fluorescence microscope in 0.04 mm^2^ areas of each retina. Cell numbers were counted manually by three people, blind to the treatments. The average density of retinal neurons per square millimeter was calculated.

### Quantification of regenerating axons

Two days before perfusion, 2 µl of CTB 647 (Thermo Fisher Scientific) was injected intravitreally. Mice were perfused with ice-cold PBS, followed by 4% PFA in 0.1 M phosphate buffer (pH 7.4). After perfusion, optic nerves were removed and postfixed in 4% PFA for overnight, and immersed in 30% sucrose overnight at 4 °C. The optic nerve was then embedded in Tissue-Tek OCT compound (Sakura Finetek, Osaka, Japan) and frozen on dry ice. Frozen sections were prepared at 14 µm using a cryostat and collected on MAS-coated glass slides (Matsunami, Osaka, Japan). For quantification, the number of CTB 647-positive axons that crossed a virtual line parallel to the lesion site at 250 µm and every 250 µm distal from the lesion site were counted manually [3], and the total number of regenerating axons were estimated by calculating the number of axons detected at a cross-sectional area of the optic nerve.

### Statistical analysis

Data are presented as means ± SEM. When statistical analyses were performed, the 1-way ANOVA followed by a Dunnett’s multiple comparison test was used. *P* < 0.05 was regarded as statistically significant. GraphPad Prism version 9.3.1 (GraphPad Software Inc., San Diego, CA, USA) was used for statistical analyses. The investigators were blinded with respect to treatment. *P* < 0.05 was considered statistically significant.

## Supplementary information

Supple files

## Data Availability

The datasets generated during and/or analysed during the current study are available from the corresponding author upon reasonable request.
