# Supplementary material for: Neuroprotection and axon regeneration by novel low-molecular-weight compounds through the modification of DOCK3 conformation
Source: Cell Death Discov. 2023 May 15;9:166. doi: 10.1038/s41420-023-01460-8 (PMC10184973; doi:10.1038/s41420-023-01460-8)
Supplement: Supplementary file 1 — Supple files [file 41420_2023_1460_MOESM1_ESM.docx]

**Supplementary Information**

Supplementary Figures S1, S2 and Figure legends

Supplementary Tables S1 and S2

**Figure S1**

**
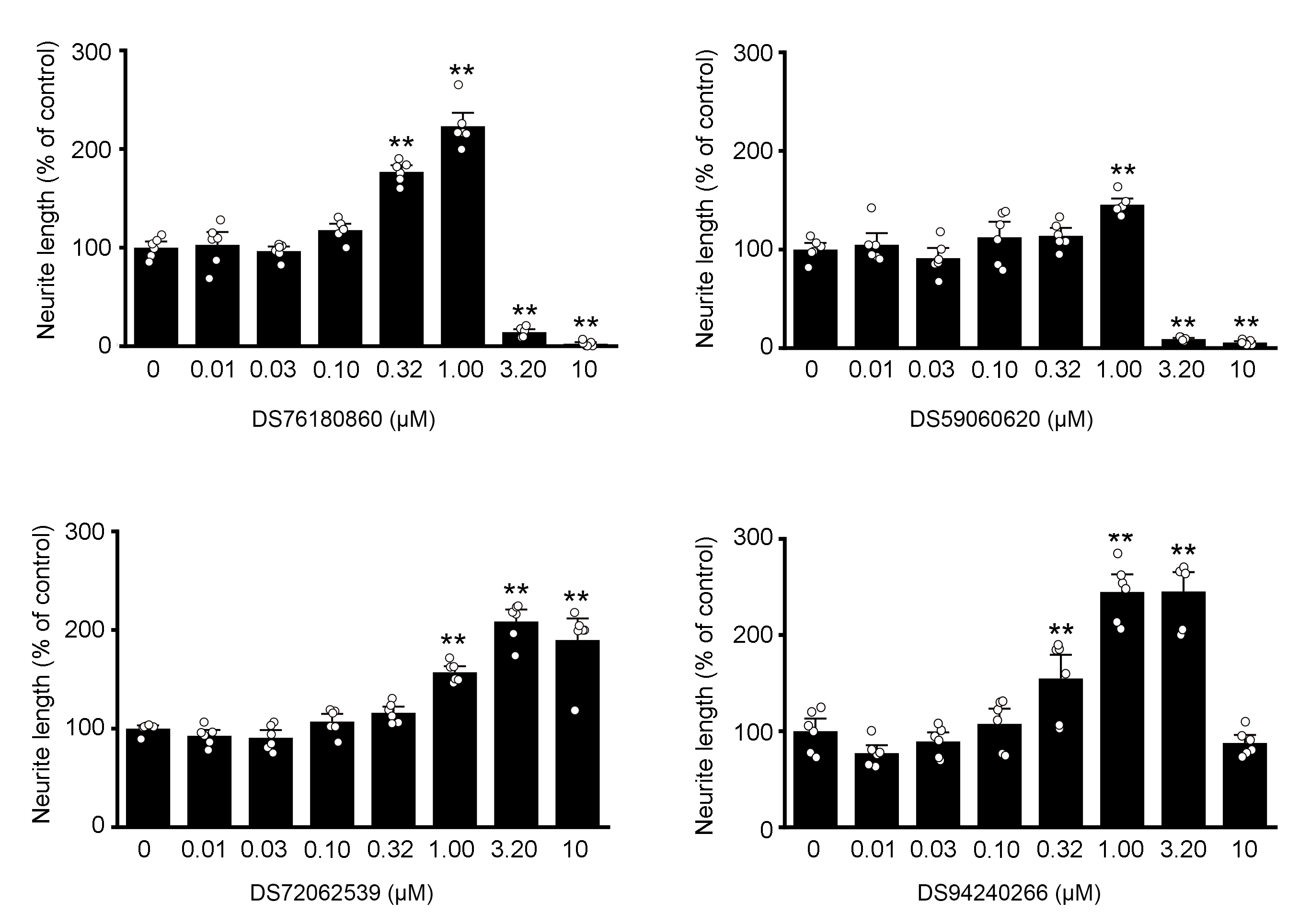
**

**The effects of the four lead compounds on neurite outgrowth in mouse cerebellum neurons.**

Primary cultured mouse cerebellum neurons were treated with each lead compound at various concentrations. Cells were fixed and stained with Tau1 antibody for detection of neurite after 2 days of culture. The length of neurite was measured by Opera Phenix. *n* = 6 per group. Data are expressed as mean ± S.E.M. ***P* < 0.01. The one-way ANOVA followed by a Dunnett's multiple comparison test was used.

**Figure S2**

**
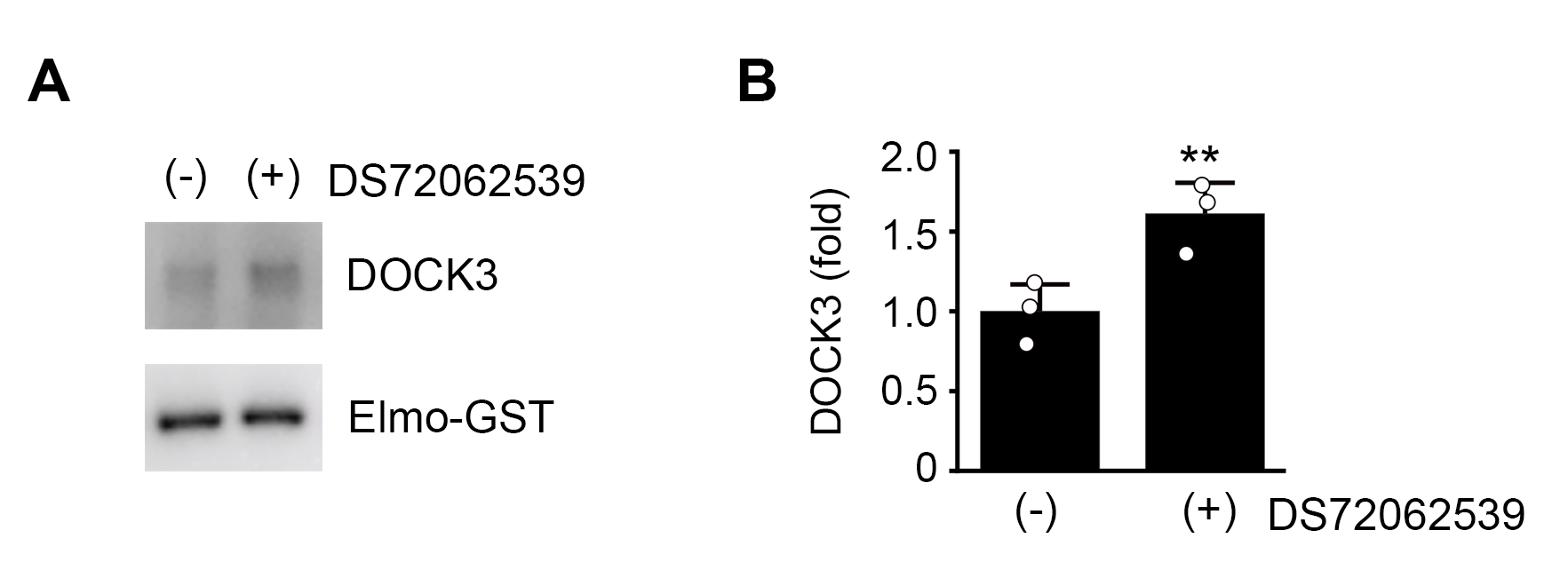
**

**The interaction between DOCK3 and Elmo was enhanced by DS72062539.**

(A) Cos7 cells were transfected with Elmo-GST plasmid followed by purification with glutathione-beads. Cell lysate from Cos7 cells transfected with DOCK3 plasmid were incubated with purified Elmo-GST in the presence or absence of DS72062539. After pull-down assay with glutathione-beads, DOCK3 bound to Elmo was detected by immunoblot analysis using a myc antibody.

(B) Quantification of the levels of DOCK3 bound to Elmo. *n* = 3 per group. Data are expressed as mean ± S.E.M. ***P* < 0.001. Student’s *t*-test was used.

**Supplementary Table S1**

Intermolecular BRET assays of the 14 primary hit compounds

| Compound ID | Signal enhancement (%) | | |
| --- | --- | --- | --- |
|  | 1 mg/mL | 5 mg/mL | 25 mg/mL |
| DS00540754 | -4.03 | 3.80 | 15.49 |
| DS02171281 | -3.96 | 0.88 | 8.77 |
| DS14111080 | -3.34 | 0.75 | 10.30 |
| DS14341381 | -4.94 | -2.19 | 14.79 |
| DS18510010 | -0.13 | 10.15 | 25.11 |
| DS19272920 | 1.23 | 6.98 | 14.73 |
| DS25961972 | 6.36 | 14.88 | 24.35 |
| DS34382322 | -3.38 | 11.10 | 19.16 |
| DS35191180 | -3.00 | 5.95 | 15.47 |
| DS43360958 | -2.44 | 5.82 | 16.33 |
| DS46450052 | -2.38 | 3.20 | 13.79 |
| DS47590519 | -0.76 | 9.96 | 22.77 |
| DS86261281 | 4.52 | 15.08 | 17.50 |
| DS88181189 | -3.49 | -0.89 | 18.17 |

**Supplementary Table S2**

Neurite outgrowth assays of the 26 derivatives of DS25961972

| Compound ID | SH-SY5Y cells | Cerebellum neurons |
| --- | --- | --- |
|  | Emax (% of BDNF) | Emax (% of DMSO) |
| DS16702826 | 107.2 | 171.5 |
| DS17040069 | 112.8 | 157.6 |
| DS21801685 | 70.3 | 179.0 |
| DS23040828 | 68.3 | 154.2 |
| DS31820013 | 94.0 | 113.9 |
| DS40741683 | 81.5 | 144.5 |
| DS45920758 | 106.9 | 163.5 |
| DS46190860 | 66.4 | 173.3 |
| DS47190860 | 101.5 | 127.4 |
| DS49851685 | 93.7 | 162.1 |
| DS49950816 | 74.0 | 108.3 |
| DS54591884 | 104.7 | 117.3 |
| DS55591884 | 112.5 | 107.9 |
| DS56240862 | 93.7 | 115.2 |
| DS59060620 | 199.8 | 145.5 |
| DS59120860 | 45.4 | 161.2 |
| DS63482426 | 54.9 | 177.4 |
| DS72062539 | 144.5 | 208.6 |
| DS73551087 | 56.7 | 121.6 |
| DS75942527 | 60.0 | 137.5 |
| DS76180860 | 46.8 | 223.1 |
| DS87851389 | 68.2 | 148.0 |
| DS94240266 | 60.3 | 245.2 |
| DS95240566 | 55.7 | 104.1 |
| DS96080827 | 91.4 | 114.0 |
| DS97170866 | 71.9 | 186.4 |
